# Bronchial artery embolization for management of massive cryptogenic hemoptysis: a case series

**DOI:** 10.1186/1752-1947-5-58

**Published:** 2011-02-10

**Authors:** Katerina D Samara, Dimitrios Tsetis, Katerina M Antoniou, Charalambos Protopapadakis, George Maltezakis, Nikolaos M Siafakas

**Affiliations:** 1 Department of Thoracic Medicine, University of Crete Medical School, Heraklion, Crete, Greece; 2 Department of Radiology, University of Crete Medical School, Heraklion, Crete, Greece

## Abstract

**Introduction:**

Hemoptysis constitutes a common and urgent medical problem. Swift and effective management is of crucial importance, especially in severe, life-threatening cases. In cases of idiopathic hemoptysis, in which no underlying pulmonary pathology can be identified, treatment is challenging. We report our experience with bronchial artery embolization in the treatment of massive idiopathic hemoptysis.

**Cases presentation:**

We report three consecutive cases of acute severe idiopathic hemoptysis. Our patients (two men aged 51 and 56 years and one woman aged 46 years), were of Caucasian ethnicity. We discuss the results and management of the patients, and review the literature. All three patients were treated safely and successfully with transcatheter embolization of the bronchial arteries using tris-acryl gelatin microspheres. Hemoptysis was controlled. All cases were followed up for 12 months, and there was no recurrence of bleeding.

**Conclusion:**

Bronchial artery embolization is an effective tool for the evaluation and treatment of massive idiopathic hemoptysis.

## Introduction

Hemoptysis is the expectoration of blood originating from the lower respiratory tract. Most cases are minor and treatable or self-limiting. The bleeding may occur from the large or small pulmonary vessels. Bleeding from the small vessels is known as diffuse alveolar hemorrhage [1]. Hemoptysis from the large vessels has multiple known etiologies, including lung neoplasms, bronchiectasis, tuberculosis, pulmonary vasculitis, cardiovascular diseases and aspergilloma. However, in a number of cases, a cause cannot be determined, and these are categorized as idiopathic hemoptysis [2,3]. The definition of severe or massive hemoptysis varies, but is usually defined as the expectoration of 300-600 ml of blood in 24 hours, or bronchial blood loss that causes hemodynamic or respiratory compromises. Hemoptysis, when severe and untreated, has a mortality rate of more than 50% [2-4]. Bronchoscopy combined with imaging technology usually identifies the bleeding site in the lungs, but in 15-20% of cases the cause of hemoptysis cannot be fully determined [3,5]. When diagnostic tools fail to identify the source of bleeding, severe hemoptysis becomes an emergency because failure to contain it can lead to death. Bronchial arteriography and bronchial artery embolization (BAE) may provide an effective means of rapid diagnosis and treatment of such medical emergencies [2,3,5].

BAE is a well-established, non-surgical procedure in the treatment of hemoptysis [3,5,6]. BAE has emerged in recent years as a treatment for severe, life-threatening hemoptysis, and has revolutionized the management of the disease, providing a reliable, minimally invasive tool with excellent diagnostic and therapeutic outcomes [3,5]. It was first described in 1973 by Remy *et al*. [7]. Subsequently, the procedure was rapidly and widely used as a treatment for severe hemoptysis, proving to be safe and efficient, and thus reducing the need for emergency thoracic surgery [8,9]. Embolization may be life-saving; it may postpone or replace surgery, and in some situations it is the treatment of choice.

## Case presentation

Three consecutive patients (two men aged 51 and 56 years and one woman aged 46 years), of Caucasian ethnicity, were treated with BAE in a tertiary academic reference center for spontaneous acute massive hemoptysis. All three were active smokers with a mean smoking habit of 50 ± 29 pack years. None had any history of chronic illness, pulmonary or otherwise. All three patients exhibited severe hemoptysis, ranging from 300 to 700 mL/day, with multiple episodes. They also had hypoxemia, anemia and low blood pressure. They were admitted to the intensive care unit for close monitoring and treatment.

Two of our patients received blood transfusions because of a rapid fall in hemoglobin levels (e.g. patient 1 had a hemoglobin level of 15.2% on admission, which had dropped to 8.7% two days later) and fear of severe hemodynamic instability,. All three of our patients were managed according to a standard hemoptysis protocol. They underwent emergency bronchoscopy and computed tomography (CT) of the thorax to identify the site of bleeding. The bronchoscopy did not allow identification of the bleeding lobe or any other significant abnormality in any of our patients. Blood trails and clots were seen in both the left and right bronchial systems, but provided no conclusive evidence as to the origin of bleeding. A tuberculin skin test and Ziehl-Neelsen examination of sputum indicated that our patients were negative for tuberculosis, and bronchial lavage cytology was negative for malignancy. The CT scan of one patient showed some degree of centrilobular emphysema. In all three cases, 'ground glass' attenuation consistent with hemorrhagic debris was found in both lungs, with predominance of one side or the other in each case. At that point, surgical management was not deemed feasible because the exact bleeding lobe could not be identified.

**Figure 1:**
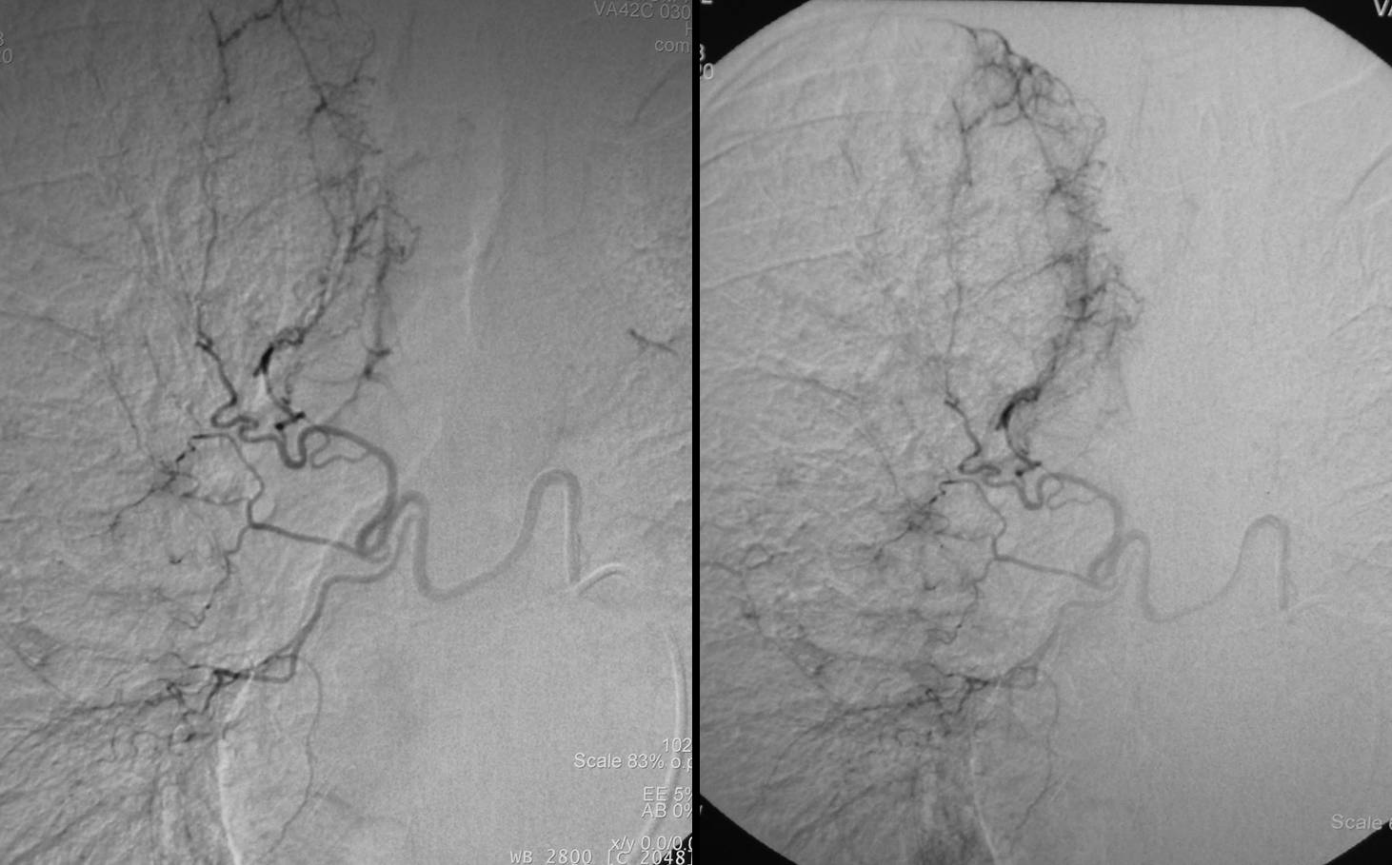
**Selective catheterization of a hypertrophic right bronchial artery in a 56-year-old man with two episodes of severe hemoptysis**. Bronchoscopy detected only some blood trails and clots in the right bronchial system, without conclusive evidence as to the origin of bleeding. Selective angiography of a hypertrophic right bronchial artery through a 5F cobra catheter demonstrates moderate hypervascularity, more prominent in the right upper lobe.

**Figure 2:**
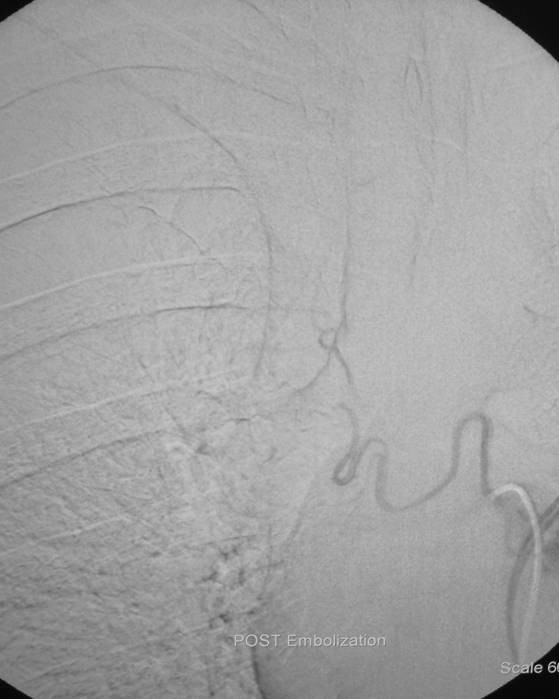
**Elimination of pathologic hypervascularity after embolization with tris-acryl gelatin microspheres (500-700 μm) injected through the cobra catheter**.

The next step was bronchial angiography followed by embolization. Under local anesthesia, the common femoral artery was percutaneously punctured, and a 5F introduction sheath was inserted. A flush catheter was advanced into the upper part of the descending thoracic aorta, and a diagnostic anteroposterior angiogram was performed, which in all three cases revealed the hypertrophic bronchial arteries. The hypertrophic bronchial arteries were then selectively catheterized with a 5F cobra-shaped curved catheter. The angiogram showed minimal to moderate hypervascularity in the right upper lobe in two cases (Figure 1), whereas in the third case, no hypervascularity or other obvious vascular abnormality was detected. Transcatheter embolization of the hypertrophic bronchial arteries of the right upper lobe was subsequently performed through the catheter after stabilization of the catheter tip was confirmed (Figure 2). A microcatheter was not used, as there was no opacification of the important spinal branches in any of our three patients. Tris-acryl gelatin microspheres (Embosphere^®^; BioSphere Medical Inc, Marlborough, MA, USA) 500-700 μm in diameter, were used as the embolization material, and were injected slowly through 1 ml syringes. The embolic particles were dispersed in contrast medium to allow visualization of any backflow and to monitor for progressive slowing of flow. Throughout the procedure, regular angiograms were performed to detect previously invisible connections to side branches supplying the spinal cord. Embolization was terminated when the antegrade flow ceased.

After the embolization treatment, all three patients were stable, and none exhibited recurrent hemoptysis. They expectorated minor amounts of blood-stained sputum, which gradually disappeared within one to three days. No complications developed in any of the cases as a result of this intervention. All three patients were discharged three to four days after embolization. Follow up CT scans at six and 12 months did not show any additional abnormality except for the aforementioned emphysema in one of the cases.

## Discussion

Life-threatening hemoptysis is one of the most serious emergencies in pulmonary medicine. The initial approach is no different than for any other bleeding or hemodynamically unstable patient. According to standard management protocols, the physician's primary goals include stabilizing the patient and securing the airway, identifying the bleeding site and efficiently containing the hemorrhage [2]. Localization of the bleeding site is usually accomplished with imaging studies (chest x-ray, CT) and bronchoscopy. In some cases, however, no underlying pulmonary pathology can be identified. When no associated comorbidity can be confidently identified, a common risk factor is cigarette smoking [10].

Management of idiopathic hemoptysis is difficult and challenging [2-4]. Surgery was once regarded as the treatment of choice in operable patients with massive hemoptysis. However, inability to localize the bleeding site makes surgery a poor option. BAE is an excellent non-surgical alternative. Indications for BAE include failure of conservative management, massive hemoptysis, recurrent hemoptysis, and elevated surgical risk. It is also done to control bleeding temporarily before surgery. According to a recent report by the Mayo clinic group [5], immediate control of bleeding is obtained in 94% of patients and 30-day control in 85% of patients. Shigemura *et al *[11] reported immediate success in controlling hemoptysis in 88% of cases in a series of 55 patients. Of those, 70% had no evidence of recurrence after one year of follow-up. It should be emphasized that after the cessation of bleeding, it is of great importance to treat any underlying pulmonary process. Another indication for BAE is peripheral pulmonary artery pseudoaneurysm, which is found in up to 11% of patients undergoing bronchial angiography for hemoptysis [12]. Although the efficacy and safety of BAE has been established in various pathologies causing massive hemoptysis, there is little information for BAE in cryptogenic hemoptysis. A recent retrospective study of cryptogenic hemoptysis in 35 smokers reported cessation of bleeding by BAE in 29 of 34 technically successful procedures (85%), and only three of five patients with recurrence of bleeding required surgical intervention [10]. Savale *et al. *[13] reported that first-line conservative measures and BAE controlled hemoptysis in 73 (90%) of their patients. Emergency surgery was performed in six patients (7%) because of failure of BAE, and secondary surgery was scheduled in a seventh patient.

BAE is described as the emergency treatment of choice for massive hemoptysis, as the mortality rate ranges from 7.1 to 18.2%, which, although high, is considerably less than the 40% seen in emergency surgery for massive hemoptysis [10,11]. Should hemoptysis recur in any treated patient, a repeat embolization can safely be performed. If the bleeding recurs one to six months later, the cause is likely to be incomplete embolization of an undetected non-bronchial systemic arterial supply. Late recurrences (6-12 months after the procedure) have been reported in as many as 2-40% of patients, probably due to disease progression [12]. Any patient with the diagnosis of cryptogenic hemoptysis has to be followed up to exclude lung carcinoma. Multidetector row CT may be helpful in this regard [13,14]. Emergency surgery for idiopathic hemoptysis should only be reserved for cases in which life-threatening bleeding continues to occur despite BAE.

Regarding the optimum embolization material for BAE, tris-acryl gelatine microspheres seem to be effective and well tolerated in patients with life-threatening hemoptysis who are not surgical candidates [15]. As has been shown in several *in vivo *and *in vitro *studies, these microspheres are characterized by better sizing and penetration characteristics than the most commonly used polyvinyl alcohol particles [16,17]. Indeed, to the best of our knowledge this report is the first to describe application of tris-acryl gelatine microspheres in consecutive patients with cryptogenic hemoptysis. The larger size particles (500-700 μm) were selected to avoid passage of the particles through bronchopulmonary shunts. We believe that further clinical and experimental studies are needed to investigate the effectiveness and safety of BAE with these particles.

BAE has proved to be very effective and lacks the mortality and morbidity related with surgical alternatives [4,11,18]. Regarding the complications of BAE, their rate has diminished gradually over the years, especially when the technique is performed by skilled and experienced radiologists. Complications include spinal cord injury, subintimal dissection of the aorta leading to mediastinal hematoma, arterial perforation by a guide wire, transient thoracic pain, shoulder pain and dysphagia [3,5]. The potential risk of spinal cord injury is the most serious complication, and must always be considered. Brown-Sequard syndrome has been reported, as has paraparesis with spontaneous regression and complete paraplegia without regression [5]. Finally, shock related to splenic infarction has been described after a successful BAE [19]. In the past few years, to prevent a potential neurologic complication developing, 'superselective' BAE has been used, meaning the embolization of more terminal branches of the arterial tree, beyond the origin of the spinal arteries. Another complication in patients with renal failure is contrast nephropathy, the risk of which must be weighed against the possible consequences, including death, of not performing BAE in a patient who cannot undergo surgery [1].

## Conclusions

We report the successful treatment by BAE of three consecutive patients presenting with cryptogenic hemoptysis. The management of hemoptysis has evolved during the past decade, favouring a least invasive therapeutic approach, as the efficacy and safety of BAE have been established for controlling hemoptysis (i.e. conservative measures and interventional radiology over emergency surgery). Our findings are in accordance with the current literature supporting BAE as a safe, non-invasive tool in the management of idiopathic bronchopulmonary hemoptysis, and advocating the use of embolization as treatment of choice in such cases. Tris-acryl microspheres appear to be a safe and effective embolization material for this application.

## Competing interests

The authors declare that they have no competing interests.

## Consent

Written informed consent was obtained from all three patients for publication of this case series and accompanying images. Copies of the written consents are available for review by the Editor-in-Chief of this journal.

## Authors' contributions

KS analyzed and interpreted patient data on the patients' disease, performed bronchoscopies and drafted the manuscript. DT performed the angiographies and bronchial artery embolizations, and was involved in drafting the manuscript. KA made substantial contributions to conception and design, and was involved in drafting the manuscript. CP participated in the acquisition and analysis of data. GM participated in the acquisition, analysis and interpretation of data. NS revised the manuscript and gave final approval of the version to be published. All authors read and approved the final manuscript.
